# Stretching oneself too thin and facing ethical challenges: Healthcare professionals’ experiences during the COVID-19 pandemic

**DOI:** 10.1177/09697330241230683

**Published:** 2024-02-05

**Authors:** Margrethe Aase Schaufel, Elisabeth Schanche, Kristine Husøy Onarheim, Ingeborg Forthun, Karl Ove Hufthammer, Inger Elise Engelund, Ingrid Miljeteig

**Affiliations:** 60518 University of Bergen; 60498 Haukeland University Hospital; 60518 University of Bergen; 25563 Norwegian Institute of Public Health; 60498 Haukeland University Hospital; 60518 University of Bergen; 60498 Haukeland University Hospital

**Keywords:** Clinical ethics, COVID-19, moral distress, professional ethics, qualitative research

## Abstract

**Backgrounds:**

Most countries are facing increased pressure on healthcare resources. A better understanding of how healthcare providers respond to new demands is relevant for future pandemics and other crises.

**Objectives:**

This study aimed to explore what nurses and doctors in Norway reported as their main ethical challenges during two periods of the COVID-19 pandemic: February 2021 and February 2022.

**Research design:**

A longitudinal repeated cross-sectional study was conducted in the Western health region of Norway. The survey included an open-ended question about ethical challenges among doctors and nurses in hospital departments. Free-text comments were analysed using Systematic Text Condensation and also presented in a frequency table.

**Ethical considerations:**

Ethical approval was granted by the Regional Research Ethics Committee in Western Norway (131,421). All participants provided consent when participating in the study.

**Results:**

In 2021, 249 and in 2022, 163 healthcare professionals responded to the open-ended question. Nurses and doctors reported three main categories of ethical challenges related to the COVID-19 pandemic: (1) barriers that hindered them in acting as they ethically would have wanted to do; (2) priority-setting dilemmas linked to overtreatment, transfer of resources and ranking patient needs; and (3) workload expansion threatening work–life balance and employees’ health. Category one comprised of resource barriers, regulatory barriers, system barriers, and personal barriers. Regulatory barriers, especially visitor restrictions for next-of-kin, were the most frequently reported in 2021. Resource barriers, related to the increased scarcity of qualified staff, were most frequently reported in 2022. Clinicians stretched themselves thin to avoid compromising on care, diagnostics, or treatment.

**Conclusions:**

Developing clinicians’ ability to handle and cope with limited healthcare resources is necessary. To foster resilience and sustainability, healthcare leaders, in collaboration with their staff, should ensure fair priority-setting and initiate reflections among doctors and nurses on what it implies to provide ‘good enough’ care.

## Introduction

A better understanding of how healthcare providers respond to new demands and limited resources is relevant for future pandemics and other crises. This study aimed to explore what nurses and doctors in Norway reported as their main ethical challenges during two periods of the COVID-19 pandemic.

## Background

The leading activity of healthcare providers is to take good care of their patients. However, various hindrances might hamper them in providing the treatment and care they regard the best, especially in low-income countries.^1^ Most clinicians in high-income settings have in the last decades been in a fortunate position where they most of the time can follow clinical guidelines for recommended treatment and care, even though priority setting has increasingly been on the agenda.^2^ Extensive healthcare budgets, a high number of trained healthcare providers, and new drugs and technology have had a significant impact on the services provided and the patient’s health and welfare.^3^ New guidelines and regulations on healthcare provision support this work, often with aims to provide good and effective care.^4^

However, handling new IT-systems, administrative tasks, and increased legal duties of documentation and transparency are some of the structural changes increasing clinicians’ workload.^5,6^ In addition, slowing economic growth, an ageing population, more patients living longer lives with severe chronic conditions and challenges related to recruiting new staff contribute to an increased burden on the healthcare system in most countries.^7,8^ A recent Norwegian Official Report stated that these problems cannot be solved solely by hiring more staff.^9^ On the contrary, each healthcare provider must be responsible for a larger number of patients without reducing the quality of care or increasing staff burnout. How this should be done is still open to discussion, and there are similar debates and processes in many countries on how to create sustainable healthcare and health workforce models.^10,11^ Despite application of new technologies and artificial intelligence, a definite solution may seem unattainable.^12,13^

The changing context of resource scarcity and the need for more regulation and priority-setting can pose new ethical challenges for healthcare providers.^14^ How will they react and respond to an increased demand to save resources, prioritize, and follow new regulations in settings with previously high access to a broad range of healthcare services? How can countries and healthcare systems provide support and prepare healthcare workers for this reality? The dilemmas arising and the responses to them during the COVID-19 pandemic might help us in answering these questions. The pandemic enforced very rapid changes upon healthcare systems and healthcare providers worldwide. Numerous studies have now been published on how healthcare providers experienced the pandemic, which ethical challenges they had to deal with, and their level of moral distress or injury.^15–19^

In a survey of nurses and doctors in Western Norway conducted in April–May 2020, we found that 67% had experienced priority-setting dilemmas the previous two weeks.^20^ Healthcare workers who were directly involved in COVID-19 care, who were redeployed, or who worked in psychiatry/addiction medicine experienced it more often. Although 59% of the respondents had seen adverse consequences due to resource scarcity, severe consequences were rare. This differed from settings where clinicians lacked ventilators and other necessary equipment to save lives.^21–23^ Reported moral distress level was generally low (2.9 on a 0–10 scale), but higher in selected groups (redeployed, managers and personnel working in psychiatry/addiction medicine). In our repeated survey of the same population in 2021 and 2022, we wanted to gain insight into healthcare workers’ experiences, perceptions, and values as the pandemic evolved. An open-ended question was therefore added to the survey to illuminate what providers found ethically demanding in periods of changing available resources, regulations, and work tasks. The overall aim was to contribute to discussions on how our healthcare system can prepare for the challenges ahead.

## Objective

The objective of this paper was to explore the ethical challenges of nurses and doctors in two different periods during the COVID-19 pandemic (February 2021 and 2022).

## Method

### Design

A longitudinal repeated cross-sectional survey was conducted in the Western Norway health region in February 2021 and February 2022. The survey included questions on demographics, occupational background, and three main themes: priority-setting dilemmas, moral distress, and employee support.^20^ As we were also interested in the health professionals’ thoughts, experiences, interactions, and motives, we included a free-text comment at the end of the survey: ‘We are interested in knowing more about which ethical challenges healthcare professionals experienced in their daily work as a consequence of the COVID-19 pandemic. Please share your experiences here (without identifiable information)’.

### Study context

The Western Norway health region (Helse Vest) covers a population of 1.1 million citizens. The yearly budget is 30 billion NOK (2.9 billion Euros), and the region consists of five local health trusts, including 50 different institutions. Respondents from four of five local health trusts in Helse Vest were included in the study: Helse Bergen, Helse Stavanger, Helse Førde, and Haraldsplass Deaconess Hospital (ideal non-profit cooperation).

### Data collection

All nurses and doctors employed in at least ≥20% positions and all managers involved in clinical work in the health trusts received a personal email invitation to participate in the study (two reminders). All hospital employees have a work email address, and the human resources department in each health trust extracted lists of employed nurses, doctors, and managers meeting the inclusion criteria. The email invitations to potential participants included information about the study, a letter of informed consent, and a personal electronic link to the online survey. In 2021, 1206 participants responded to the survey, of whom 249 responded to the open-ended question about experienced ethical challenges. The numbers in 2022 were 682 and 163, respectively. Demographics are presented in Table 1.

**Table 1.** Respondent descriptive characteristics (*n* = 372).

	2021 (*n* = 249)	2022 (*n* = 163)
Age		
20–29 years	22 (8.8%)	6 (3.7%)
30–39 years	57 (23%)	42 (26%)
40–49 years	74 (30%)	47 (29%)
50–59 years	63 (25%)	44 (27%)
60+ years	33 (13%)	24 (15%)
Gender		
Female	194 (78%)	126 (77%)
Male	55 (22%)	36 (22%)
Other	0 (0%)	1 (0.6%)
Main position		
Specialist nurse	105 (42%)	66 (40%)
Nurse	56 (22%)	35 (21%)
Consultant (senior doctor)	61 (24%)	43 (26%)
Registrar (doctor under specialisation)	15 (6.0%)	11 (6.7%)
First-year resident	3 (1.2%)	0 (0%)
Other	9 (3.6%)	8 (4.9%)
Health trust/hospital		
Haukeland University Hospital	120 (48%)	76 (47%)
Stavanger University Hospital	98 (39%)	72 (44%)
Førde Central Hospital	14 (5.6%)	9 (5.5%)
Haraldsplass Deaconess Hospital	17 (6.8%)	6 (3.7%)
Department		
Medical specialities	84 (34%)	52 (32%)
Surgical specialities	39 (16%)	43 (26%)
Anaesthesia or intensive care medicine	71 (29%)	30 (18%)
Psychiatry or addiction medicine	46 (18%)	28 (17%)
Other	9 (3.6%)	10 (6.1%)
Time employed		
<1 year	4 (1.6%)	0 (0%)
[1, 4) years	33 (13%)	21 (13%)
[4,10) years	52 (21%)	37 (23%)
[10,20) years	80 (32%)	53 (33%)
20+ years	80 (32%)	52 (32%)
Managerial responsibilities	51 (20%)	40 (25%)
Directly involved in treatment or care of COVID-19 patients	95 (38%)	94 (58%)
Redeployed or given other tasks due to COVID-19	78 (31%)	52 (32%)

### Analysis

Qualitative analysis was conducted in collaboration by the authors MAS, IM, ES, and KHO following Systematic Text Condensation, a cross-case thematic analysis.^24^ Analysis proceeded through the following stages: (i) reading all the material to obtain an overall impression, bracketing previous preconceptions; (ii) identifying units of meaning, representing different aspects of the participants’ experiences of ethical challenges and coding for these; (iii) condensing and abstracting the meaning within each of the coded groups; and (iv) summarizing the contents of each code group to generalized descriptions and concepts reflecting the most important aspects of ethical dilemmas reported by the respondents.

The analysis was done stepwise, starting with material from 2021, and then adding the material from 2022. A decision trail^25^ documented the choices during the analytical process, using an editing analysis style.^26^ This method combines inductive and deductive analysis. Categories are mainly inductively developed from the data. Qualitative analysis is influenced by the researchers’ preconceptions and theoretical framework, and the deductive element involves applying this framework and the research question to identify categories. The research team consisted of doctors (MAS, IM, and KHO), ethicist (IM), psychologist (ES), sociologist (IEE), economist (IF), and statistician (KOH), providing different angles and interdisciplinary discussions on the topics of investigation. Inspired by the theory of moral distress and resilience, the findings reflect different aspects of these concepts^27^ but are not organized in a theory-driven template analysis style.

### Ethical considerations

Ethical approval was granted by the Regional Research Ethics Committee in Western Norway (131,421). All participants provided consent when participating in the study. [add more detail regarding confidentiality/anonymity and support for participants].

## Results

Nurses and doctors reported three main categories of ethical challenges related to the COVID-19 pandemic: (1) barriers that hindered them in acting as they ethically would have wanted to do; (2) priority-setting dilemmas linked to overtreatment, transfer of resources, and ranking patient needs; and (3) workload expansion threatening work–life balance and employees’ health.

Below, we elaborate on these findings, supported by quotations from the participants.

### Barriers to adequate treatment, care, and follow-up

**Figure 1.**
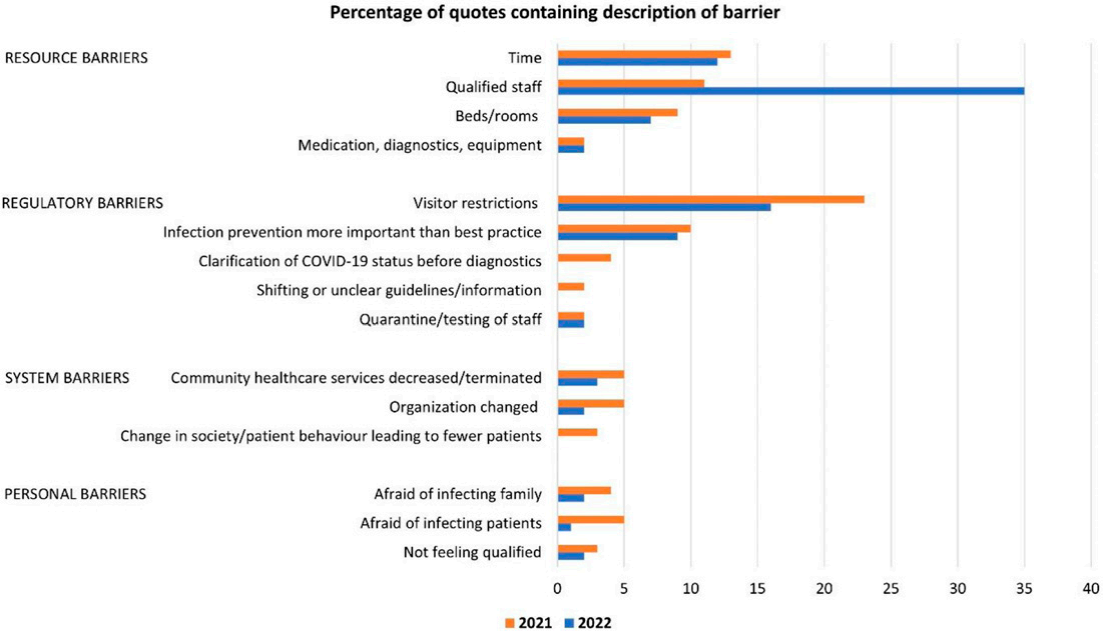
Percentage of quotes containing description of barriers.

Most of the nurses and doctors described situations where they were hindered in doing what they regarded as best practice in patient care or diverging from the treatment and care they provided before the COVID-19 pandemic. The described barriers could be divided into four types, with several sub-types (Table 2). Resource barriers were time hindrances, scarcity of qualified staff, beds, and rooms, and a few respondents also reported a lack of equipment or diagnostics. No one reported a scarcity of drugs. Quotes mentioning barriers were 259 in 2021 and 122 in 2022. Figure 1 shows the frequency of quotes containing descriptions of the type of barriers. Regulatory barriers, especially the visitation restrictions for next-of-kin, were the most reported barrier in 2021. Resource barriers related to the increased reported scarcity of lack of qualified staff were the most frequent reported barriers in 2022.

**Table 2.** Categorization of the responses according to the four categories of barriers to adequate treatment, care, and follow-up from 259 participants in 2021 and 122 participants in 2022. The quotes were coded according to the main barrier described.

Barriers to adequate treatment, care, and follow-up	
Sub-category	Type of barrier
Resource barriers	Time
	• More patients, more time-consuming procedures without extra staff
	• Reduced efficiency due to new routines, restrictions, and untrained staff
	• Busy – multitasking, interrupted, and intensified work
	Qualified staff
	• Lack of qualified staff due to quarantine or sick leave
	• Lack of specially trained staff for new practice/treatment of patients
	• Increased pressure on the staff at work
	Beds/rooms
	• Reduced number of beds
	• More pressure on the beds available
	• Higher turn-over
	Medication, diagnostics, and equipment
Regulatory barriers	Visitor restrictions for next-of kin
	Infection prevention is more important than best care and practice
	Clarification of COVID-19 status of patients before diagnostics etc.
	Shifting or unclear guidelines and information
	Quarantine/testing of staff
System barriers	Community healthcare services decreased or were terminated
	Organization changed
	Change in society/patient behaviour leading to fewer patients seeking care
Personal barriers	Afraid of infecting family
	Afraid of infecting patients
	Not feeling qualified for the job

Lack of time was a crucial factor. New or more simultaneous duties related to COVID-19 entailed less time for each patient. Infection prevention was time-consuming, as was contingency planning. Sick leave, allocation of staff, and lack of temporary employees made it very difficult to maintain adequate competence and sufficient capacity among staff on the wards, especially in 2022. When the number of patients increased, lack of resources challenged patient safety and adequate follow-up. Participants outlined how patients with unstable medical conditions did not receive sufficient monitoring, and patients were discharged too early in their treatment course due to a lack of beds. Communication with patients and relatives was forced to a minimum. Healthcare professionals described how resource constraints led to less efficient care, for instance, when there were not enough doctors present to discharge patients or the doctors present were not familiar with the patient group. Some had experienced a lack of equipment, but the main challenge was the lack of staff:

“The staffing is so poor that the patients who ‘scream the loudest’ often are the ones who get help, while those who are more modest lie and wait. I often experience that quite a lot of what should have been done does not get done because we’re in a hurry. Constant disruptions and interrupted work tasks make everything take longer and be less safe.” (Participant 131)

One of the most burdensome challenges reported by the participants was linked to their new roles in enforcing COVID-19 guidelines or indirect effects on other healthcare services. They experienced profound negative effects of these restrictions for both patients and relatives, contrasting their moral imperative of doing good. Several respondents expressed disagreement with COVID-19 regulations separating family members from a dying person, a father from his new-born child, or themselves from children and adolescents in need of psychological therapy. Witnessing how the regulations affected patients and relatives was hard, and some reported feelings of shame when providing what they regarded as undignified care. Other hindrances were reduced healthcare services in the municipalities, especially in the fields of psychiatry, addiction medicine, and rehabilitation, which disrupted adequate follow-up and caused reduced quality of life. Sometimes hospital stays were prolonged because of this barrier, which in turn diminished the overall capacity of the hospital to receive new patients with acute illness. Video and telephone consultations were regarded as unsuitable for many patient groups, even though it was recommended by the authorities. Several participants described how they had to perform tasks without the necessary qualifications because of resource scarcity. This could result in postponed discharge when doctors were unfamiliar with the patient group or hired personnel not knowing basic routines. However, the most emphasised hindrance was the dilemma caused by measures on infection prevention:

“[I experienced] dilemma around safeguarding infection control and at the same time maintaining the quality of psychiatric treatment. Difficult to place restrictions on disorientated patients who do not understand the basis for these [restrictions] and are unable to comply with guidelines. Some measures can slow down rehabilitation (impeding socialization and training in various skills), weaken the relationship with helpers (fewer conversations/meeting points/long distance/video use), or lead to premature discharge because patients are unable to adhere to a minimum of infection control rules/ show consideration for fellow patients and staff. More difficult to work at a greater physical distance from colleagues – which reduces cooperation and training of new staff, becomes more tired yourself.” (Participant 89)

The COVID-19-specific visitor regulations were reported as very difficult to handle. It was hard to be the one to decide who and for how long relatives of severely ill patients were allowed to enter the hospital. This caused moral distress, and they had to face the sorrow and anger of both patients and relatives. Some expressed it as heart-breaking to deprive patients of the support they needed from their loved ones, such as breaking bad news to patients without family members or friends present. They also noted that this had clinical implications in terms of depression and worsened quality of communication. This also added to healthcare professionals’ workload, as they spent time explaining restrictions on the phone and dealing with complaints and frustrations. It was difficult for healthcare workers to adapt to this new role and find good solutions to the situations that occurred when COVID-19 restrictions hampered their possibility to act according to what they regarded as best clinical practice.

“I find it burdensome to be a ‘watchdog’ towards patients and relatives. Constantly had to explain, guide, and refuse due to COVID-19 restrictions. The patients are not allowed to go on trips, leave home, or visits which are an important part of the treatment.” (Participant 69)

### Priority-setting dilemmas linked to overtreatment, transfer of resources and ranking patient needs

Healthcare personnel described that unnecessary and unethical treatment was provided, such as continuing intensive care for COVID-19 patients with a bleak prognosis. This was experienced as working against their conscience. Some mentioned a substantial risk of overtreatment because of prognostic uncertainty related to the new disease. Care for patients who were severely affected by COVID-19 but had serious underlying illnesses and yet received treatment such as respiratory support, was described as ethically challenging. Several pointed at a practice of so-called ‘defensive medicine’, with frequent overdiagnosis and overtreatment. This was not noted as COVID-19-specific but as a continuation of a broader tendency that has developed over the past years. This general tendency comprised unrealistic and increasing expectations and demands from patients, while healthcare personnel thought treatment should stop earlier. Informants elaborated on the difficulty of witnessing patients receiving burdensome therapy at the end of life because the family opposed withdrawing life-sustaining treatment. This was found to be ethically problematic and undignified for the patient. The patients experienced more complications than benefits because of overtreatment, especially among COVID-19 patients with a high body mass index.

“Weeks and weeks of intensive care treatment of patients with a dismal prognosis. I have experienced this as purely unethical, and I had to work against my conscience.” (Participant 35)

Healthcare workers found it ethically challenging that resource-demanding COVID-19 treatment was prioritized over other patients who needed help. This included patients of all age groups. Clinicians outlined how frail and older patients with acute or chronic functional impairment did not receive the examination or services they needed. This was rooted in a lack of resources in the departments, with healthcare personnel who were absent due to illness or COVID-19 testing, reduced visitor services, and limited services for discharge to municipality care after hospitalization. Participants described how patients who had previously benefited from low-threshold services such as day centres, visits from relatives, and dementia teams no longer had access to these services. Their cognitive and physical function was weakened, and loneliness increased. There were examples of patients being discharged too early and others too late, without adequate follow-up and support in place. At the other end of the age scale, they saw families who were struggling at home with their children, where the child or adolescent was waiting for elective examination, diagnosis, and treatment in psychiatric healthcare. It was described as ‘horrific’ and reprehensible to reduce the healthcare services for these families in favour of prioritizing COVID-19 efforts. Participants experienced that the families were left behind because staff were redeployed to other departments to take care of emergency functions. De-prioritization of non-COVID-19 illness also affected cancer patients. This became a vicious circle according to one participant:

“We see the consequences for all cancer patients who have waited a long time for surgery due to postponements because of COVID-19. The patients are worse off postoperatively and have more complications, which in turn leads to more days in bed, which in turn can delay other cancer patients waiting for surgery, as we must cancel operations due to poor capacity.” (Participant 194)

Participants conveyed that the ‘unfair’ consequences particularly applied to patients with non-fatal diseases, who had operations and other treatments postponed, causing considerably reduced quality of life. Examples comprised patients with endometriosis suffering from pain and patients with troublesome prolapse, who instead of 6 months had waited 12 months for an operation and then were postponed again. They found it tiring to inform patients that their surgery would be postponed because the postoperative services were unavailable due to the increased need for intensive care services for COVID-19 patients. Healthcare personnel did however understand the need for priority-setting, but outlined the suffering caused by delayed treatment for their patients as ethically challenging:

“The consequences for these patient groups are that they have to live with pain, are more exposed to infections, are on sick leave as a result of pain, as well as the psychological aspect of waiting for surgery without being assigned a new surgery date.” (Participant 271)

### Workload expansion threatening work–life balance and employees’ health

The participants described how they mobilised all their energy and effort to compensate for the restrictions and assist their colleagues in helping the patients. This caused a lot of strain, including overtime and extra shifts, often combined with domestic responsibilities, like home school and home nursery. Some expressed how they would never be able to endure this load again. They went to great lengths to cover shifts and felt pressured to take extra or double shifts due to the increased need to take care of critically ill patients. Even if they had symptoms and were not well, they felt they had to go to work, because they knew the increased burden on their colleagues if they did not show up. The remaining staff experienced a heavy workload when many colleagues were isolated with COVID-19 disease or in quarantine because they were close contacts. There were many concurrent conflicts, and important tasks were pushed aside due to a lack of resources. They described dilemmas that involved having to push themselves even further to satisfy the patients’ needs and provide adequate care. Although they stretched themselves thin, feelings of inadequacy remained, and they ‘just had to keep on working their way through the days’, noting the potential high personal cost for themselves and a blurred work–life balance.

“The ethical dilemma lies within me: should I let the patient wait and possibly let their condition deteriorate, or should I work overtime again, hoping to help the patient, but then perhaps it affects myself? In the end, I get sick and can’t help anyone. It usually affects me! I cannot stand to keep the patient waiting. I haven’t become sick yet, but it’s not far from it.” (Participant 48)

Participants spread themselves thin because of the pressure from the department and management. Increased workload and increased pace after the first COVID-19 waves were referred to by some as the department ‘making up for lost time’. Some stated that the main challenge they faced was the exploitation of employees, who experienced significant stress and pressure related to the high number of tasks and pace during the working day, with few or no breaks, and pressure from the employer. Even though several employees were sick or in quarantine, some experienced that the demands from the management on what the unit should be able to do kept increasing. Another strain was related to an expanding responsibility for difficult decisions, where the consequences of ‘wrong’ choices in some cases could be fatal:

“Being accountable for the decision of granting leave – if infection were to be brought into the ward, and at the same time also risking being accused of worsening the patients’ disorder or causing premature discharge resulting in subsequent suicide or homicide.” (Participant 122)

On the other hand, managers described how challenging it was to run the departments in a good way during the pandemic. They also stretched themselves to compensate for the dilemmas the COVID-19 setting posed. It was difficult to prioritize training and follow-up of new personnel in the department, and they reported how they were unable to follow up tired colleagues well enough due to the high work pressure. The new employees needed to become competent even faster than before, but a manager also described the need for them to become confident in themselves and felt that extra time should be spent on support and training. At the same time, there were more managerial tasks than usual, and it was impossible to handle these without a lot of overtime. Feeling responsible for employees who were worried and afraid was reported as stressful, as was being in charge in a time of uncertainty. A manager describes how this also affected his/her state of health:

“As a manager, it has been burdensome to feel responsible for employees who have been worried and afraid. During the ongoing outbreak, it has been demanding to be a leader due to a lot of uncertainty regarding the further development of the situation. It has affected my health in the form of poor sleep, reduced food intake, stress, and anxiety.” (Participant 146)

## Discussion

This study demonstrates how nurses and doctors in a high-income country like Norway were put under stress during the COVID-19 pandemic. They experienced ethical challenges, resource constraints, and barriers that hindered them from acting according to their moral standards. The most common hindrances varied between 2021 and 2022. The participants reported ethical dilemmas linked to overtreatment and transfer of resources, and that their work–life balance was compromised. Below, we discuss the implications of our findings in the light of previous research, which ethical principles are at stake, and how these are relevant to developing resilient health systems, preparedness, and response capacity.

### Moral principles meet reality

The hindrances and compensatory mechanisms in response to ethical challenges in this study revealed conflicting ethical principles and values. A common way to analyse what is at stake in clinical ethical dilemmas is to use the four principles approach, looking at conflicts and trade-offs between the principles of beneficence, nonmaleficence, respect for patient autonomy, and justice.^28^ A central conflict was the dilemmas between the principles of doing good and hindering harm.^28^ For instance, participants highlighted the dilemma around safeguarding infection control and at the same time maintaining the quality of psychiatric treatment. The challenges of visitor guidelines, which implied restricting visitors to severely sick patients, were expressed as ‘heart-breaking’. To protect people from potentially life-threatening infections, they deprived patients of the support they needed from their families, knowing that this also had clinical implications in terms of depression and reduced quality of life. Ensuring dignity was among the values being threatened, for instance, when providing burdensome therapy which was regarded futile by the clinicians. This could be experienced as dignity violation,^29^ as opposed to the standards of dignity conserving care.^30^ Our findings align with previous research demonstrating difficulties in assessing end-of-life needs and providing palliative care during the pandemic.^31^ The solidarity and societal responsibility conflicted with concerns about what would be best for the patient. Respecting patient autonomy was highlighted as paramount, and not being able to adhere to this principle may violate healthcare professionals’ moral agency and virtues like compassion, integrity, and conscientiousness.^32,33^ Justice, in the sense of fair, equitable, and appropriate distribution of healthcare resources,^28^ was another value they saw being compromised. Some of them experienced their patient population almost overnight being assessed as ‘less worth’ due to the overall priority of COVID-19 patients and related restrictions. There were numerous examples of responders who found it unfair that children, families, and elderly patients experienced increased or prolonged suffering from psychological neurological, oncological, or gynaecological disorders because resources were used to provide care for COVID-19 patients. In addition, they reported an unfair pressure towards stretching themselves thin and risking their health for the sake of the patients. As emphasised by Palmer and colleagues, there is a need to address contributors to moral distress both at the individual, system, and situation levels, and develop interventions to mitigate negative outcomes in future healthcare crises and daily clinical practice.^34^

### Professional identity under pressure

This study revealed that healthcare professionals’ identity was challenged by their new roles, especially when executing guidelines with negative consequences for their patients and relatives. Healthcare providers described it as burdensome to be a ‘watchdog’ towards patients and relatives instead of advocating for their rights and benefits. Doctors in Norway, especially general practitioners, are familiar with the task of sometimes denying patients’ requests due to the responsibility of avoiding futile treatment and undue spending of public resources.^35^ Healthcare professionals may develop different strategies to execute their gatekeeping role and at the same time attend to patients’ needs and wishes.^36^ Tong and colleagues showed that even though nurses experienced stress and challenges when coping with new tasks, caring for patients with COVID-19 could be associated with a stronger professional identity, especially a ‘sense of self-potency’.^37^ Moula and colleagues studied medical students’ views on their professional role and described a tension between their sense of duty and sense of well-being, as well as an increased awareness of the social and global role of doctors in addressing health inequities.^38^ They also became more aware of their personal needs, priorities, and the importance of self-care. During the pandemic, both healthcare professionals and managers had to be responsible for difficult decisions in a context where they lacked comparable experiences. Thus, the long-term consequences or outcome of their decisions were highly uncertain. This uncertainty can be likened to having a lack of control, which is thought to result in job strain. Prolonged job strain may contribute to burnout, mental and physical illnesses, ineffective performance, absenteeism, reduced organizational commitment, and turnover.^39,40^ We believe that increased attention towards a sustainable professional identity as part of healthcare professional education may equip future nurses and doctors with tools and coping strategies necessary to meet the challenges they will face. In particular, there is a need to provide frameworks for and training in how to handle uncertainty in medical practice.^41^

### How can experiences from the COVID-19 pandemic guide future practice?

The findings from this study highlight the impact of societal and clinical guidelines and the conflicts experienced when using these in complex everyday settings. Bringedal and colleagues studied general practitioners and hospital doctors’ assessment of priority guidelines in Norway during the COVID-19 pandemic. They concluded that healthcare providers may find it hard to accept rationing of care in general, especially while observing vulnerable patients being deprioritized.^42^ The pandemic crisis may however also stimulate constructive ways of preparing for challenges ahead, like increased cross-sectional and interdisciplinary collaboration and innovation.^43^ Studies from the COVID-19 pandemic found that facing ethical uncertainty and end-of-life challenges may install growth and adjustments that can serve as inspiration and organizational change.^44,45^ This study pointed at the practice of so-called ‘defensive medicine’ and described overdiagnosis and overtreatment for COVID-19 as part of an increasing tendency. Overtreatment is an ethical challenge worldwide driven by many inter-related factors,^46^ and efforts like the ‘Choosing Wisely’ campaign are among interventions that could counteract this unwanted development.^47^ Supporting healthcare professionals in decision-making when they regard treatment should be discontinued earlier is crucial, especially when facing shrinking healthcare budgets, fewer healthcare workers, and older populations. This study, from a pandemic, demonstrates that the ethical burden of handling such situations can be high on healthcare workers. Looking ahead it remains an unanswered question whether adaption to resource constraints inevitably implies lowering healthcare professionals’ moral or medical standards. If so, this dilemma deserves further study and public and professional discussion. Restructuring of clinical practice to the changing healthcare system during COVID-19 also demonstrates possibilities of improved rather than decreased quality of care.^48^ To decrease burdensome therapy at the end of life, as some of our participants reported in our study, efforts should be made to integrate interventions enabling healthcare professionals to deal with complex decision-making in life-threatening diseases ^49^ and promote end-of-life discussions.^50^ Ethical reflection groups, like moral case deliberation,^51^ clinical ethics committees or consultations, development of ethical guidelines for withholding and withdrawal of life support, and initiatives to Advance Care Planning and frailty assessment should be explored further as possible initiatives.

### How can the COVID-19 pandemic foster moral resilience?

This study confirms previous research demonstrating how nurses and doctors suffered from moral distress during the COVID-19 pandemic.^52,53^ In addition, our findings illuminate how experiences of barriers and moral distress differed between 2021 and 2022. Regulatory barriers, especially the visitor restrictions for next-of-kin, were the most reported barrier in 2021. In 2022, visitor restrictions were repealed, and more healthcare professionals were on sick leave. Thus, resource barriers due to a lack of qualified staff were the most frequently reported barrier in 2022. This underlines that system barriers and resource constraints are important contributors to moral distress. The high moral standards of healthcare professionals in this study may have diminished additional severe consequences of the pandemic, in terms of working harder and suppressing personal needs to help a larger number of patients. The strategy of stretching themselves thin when facing these challenges, as reported by participants in our study, is well-intended, but not a sustainable coping mechanism. From the onset of the pandemic, healthcare providers have also reported ways of coping more fruitfully with the dramatic changes they experienced. Facing ethical challenges on multiple fronts, nurses have been able to find ways of solving problems, achieve role adaptation, reduce ethical conflict, and identify and deal with unhealthy emotions.^54^ Formative experiences gained during the pandemic have influenced doctors’ end-of-life decisions and increased their focus on factors affecting treatment outcomes like age and frailty in addition to resource limitations. Healthcare professionals in this study reported ethical challenges linked to overtreatment and working against their conscience. Chang and Matthews found that over half of clinicians in their survey reported that they more often assessed that patients should not be resuscitated than pre-pandemic. A sizeable minority reported that they now have a higher threshold for referring to intensive care units, and a lower threshold for palliation.^55^ Our findings underline the importance of supporting clinicians to adapt to the current situation of an increasing elderly population in need of healthcare when resources are not expected to increase accordingly. Ensuring fair priority-setting and being able to provide ‘adequate’ care will be key, and doctors and nurses will often be the ones taking overall principles and guidelines into everyday practice. To support this, healthcare leaders and sub-level leaders should in collaboration with their staff start the work to foster resilience. Clinical ethics support can facilitate awareness, skills, and knowledge related to fairness concerns, trade-offs of values, and strategies to ensure legitimate processes, as well as support staff in tough decisions.^56^ Other interventions cultivating moral resilience have been outlined by Rushton and colleagues, for example, helping staff recognize moral distress responses and various coping mechanisms, increasing their ethical competency and confidence in dealing with complex situations.^27^ Reducing one’s expectations regarding possible achievements given the circumstances is emphasised to develop resilience, as opposed to the strategy of self-suppressive overcompensation expressed by our participants.

### Strengths and limitations

Free-text comments as part of standardized questionnaires can provide additional perspectives from participants on the topic under investigation. Informants having very good or very poor experiences may be more likely to provide qualitative free-text responses. Further, healthcare professionals experiencing hectic workdays may not have had time to participate in the study, which could have impacted the data material. Still, valuable knowledge can be obtained which otherwise would have been missed in solely quantitative data collection.^57,58^ The timing and context of our data collection influenced the findings and must also be taken into account when comparing the results with other studies. We have read with great precaution the results in publications on the ethical challenges and increased moral distress among healthcare providers during the COVID-19 pandemic. We acknowledge that the pandemic was extremely hard for many. Still, we also question the conclusions of some of these studies that do not compare their responses over time, have few responders, and are prone to selection bias.

An interdisciplinary research team provided different perspectives during the planning of the study and analytical process, illuminating medical, ethical, psychological, social, and economic angles. However, researchers with other professional backgrounds and theoretical lenses could have pursued additional aspects of the free-text comments. This study comprised a varied sample of participants recruited from a broad spectrum of medical and nursing specialities, adding transferability and relevance for other similar settings. Since Norway is a high-income country with universal health coverage, our findings may not be transferable to countries with different healthcare organizations and fewer resources.

## Conclusions

In a high-income country like Norway, the COVID-19 pandemic caused ethical challenges among healthcare providers in terms of barriers to what they regarded as best clinical practice, priority-setting dilemmas, and exhausting overcompensation to protect patients and colleagues. Efforts to strengthen health system resilience to meet new crises and in response to expected burdens on the health systems must support healthcare providers’ moral resilience and develop sustainable professional identities accustomed to resource constraints. Ensuring fair priority-setting and being able to provide ‘good enough’ care is key for doctors and nurses, and healthcare leaders should address this in collaboration with their staff to foster resilience.

## References

[1] Defaye FB Desalegn D Danis M , et al. A survey of Ethiopian physicians' experiences of bedside rationing: extensive resource scarcity, tough decisions, and adverse consequences. BMC Health Serv Res 2015; 15: 467. DOI: 10.1186/s12913-015-1131-6. 26467298 PMC4607248

[2] Seixas BV Regier DA Bryan S , et al. Describing practices of priority setting and resource allocation in publicly funded health care systems of high-income countries. BMC Health Serv Res 2021; 21(1): 90. DOI: 10.1186/s12913-021-06078-z. 33499854 PMC7839200

[3] Mahara G Tian C Xu X , et al. Revolutionising health care: exploring the latest advances in medical sciences. J Glob Health 2023; 13: 03042. DOI: 10.7189/jogh.13.03042. 37539846 PMC10401902

[4] D’Andreamatteo A Ianni L Lega F , et al. Lean in healthcare: a comprehensive review. Health Pol 2015; 119(9): 1197–1209. DOI: 10.1016/j.healthpol.2015.02.002. 25737260

[5] Yan Q Jiang Z Harbin Z , et al. Exploring the relationship between electronic health records and provider burnout: a systematic review. J Am Med Inf Assoc 2021; 28(5): 1009–1021. DOI: 10.1093/jamia/ocab009. PMC8068439 33659988

[6] Thun S Halsteinli V Løvseth L . A study of unreasonable illegitimate tasks, administrative tasks, and sickness presenteeism amongst Norwegian physicians: an everyday struggle? BMC Health Serv Res 2018; 18(1): 407. DOI: 10.1186/s12913-018-3229-0. 29871623 PMC5989409

[7] Cook C Cole G Asaria P , et al. The annual global economic burden of heart failure. Int J Cardiol 2014; 171(3): 368–376. DOI: 10.1016/j.ijcard.2013.12.028. 24398230

[8] Marc M Bartosiewicz A Burzynska J , et al. A nursing shortage - a prospect of global and local policies. Int Nurs Rev 2019; 66(1): 9–16. DOI: 10.1111/inr.12473. 30039849

[9] NOU 2023: 4 . Tid for handling — Personellet i en bærekraftig helse- og omsorgstjeneste. [Time for action – Workforce in a sustainable healthcare system] (In Norwegian). Sacramento, CA: Ministry of Health and Care Services, 2023. NOU 2023: 4 - regjeringen.no (accessed 23.06.23).

[10] Holmer S Nedlund AC Thomas K , et al. How health care professionals handle limited resources in primary care - an interview study. BMC Health Serv Res 2023; 23(1): 6. DOI: 10.1186/s12913-022-08996-y. 36597086 PMC9808951

[11] Engelseth P Kozlowski R Kamecka K , et al. Framing sustainable healthcare services. Int J Environ Res Publ Health 2021; 18(12): 6336. DOI: 10.3390/ijerph18126336. PMC8296158 34208128

[12] Figueroa CA Harrison R Chauhan A , et al. Priorities and challenges for health leadership and workforce management globally: a rapid review. BMC Health Serv Res 2019; 19(1): 239. DOI: 10.1186/s12913-019-4080-7. 31014349 PMC6480808

[13] Meskó B Hetényi G Győrffy Z . Will artificial intelligence solve the human resource crisis in healthcare? BMC Health Serv Res 2018; 18(1): 545. DOI: 10.1186/s12913-018-3359-4. 30001717 PMC6044098

[14] Haahr A Norlyk A Martinsen B , et al. Nurses experiences of ethical dilemmas: a review. Nurs Ethics 2020; 27(1): 258–272. DOI: 10.1177/0969733019832941. 30975034

[15] Spilg EG Rushton CH Phillips JL , et al. The new frontline: exploring the links between moral distress, moral resilience and mental health in healthcare workers during the COVID-19 pandemic. BMC Psychiatr 2022; 22(1): 19. DOI: 10.1186/s12888-021-03637-w. PMC8734541 34991514

[16] Holtz HK Weissinger GM Swavely D , et al. The long tail of COVID-19: implications for the future of emergency nursing. J Emerg Nurs 2023; 49(2): 198–209. DOI: 10.1016/j.jen.2022.10.006. 36503829 PMC9584853

[17] Mejdahl CT Nielsen BK Mehlsen MY , et al. COVID-19 as moral breakdown: entangled ethical demands experienced by hospital-based nurses in the early onset of the pandemic. Nurs Inq 2023; 30(1): e12508. DOI: 10.1111/nin.12508. 35709227 PMC9349400

[18] Rushton CH Thomas TA Antonsdottir IM , et al. Moral injury and moral resilience in health care workers during COVID-19 pandemic. J Palliat Med. 2022; 25(5): 712–719. DOI: 10.1089/jpm.2021.0076. 34678091 PMC9081047

[19] O'Mathúna D Smith J Zadvinskis IM , et al. Ethics and frontline nursing during COVID-19: a qualitative analysis. Nurs Ethics 2023; 30(6): 803–821. DOI: 10.1177/09697330221143150. 36971185 PMC10051012

[20] Miljeteig I Forthun I Hufthammer KO , et al. Priority-setting dilemmas, moral distress, and support experienced by nurses and physicians in the early phase of the COVID-19 pandemic in Norway. Nurs Ethics 2021; 28(1): 66–81. DOI: 10.1177/0969733020981748. 33430698 PMC7879232

[21] Truog RD Mitchell C Daley GQ . The toughest triage – allocating ventilators in a pandemic. N Engl J Med 2020; 382: 1973–1975. 32202721 10.1056/NEJMp2005689

[22] Kirkpatrick JN Hull SC Fedson S , et al. Scarce-resource allocation and patient triage during the COVID-19 pandemic: JACC review topic of the week. J Am Coll Cardiol 2020; 76(1): 85–92. DOI: 10.1016/j.jacc.2020.05.006. 32407772 PMC7213960

[23] Maffoni M Fiabane E Setti I , et al. Moral distress among frontline physicians and nurses in the early phase of COVID-19 pandemic in Italy. Int J Environ Res Publ Health 2022; 19(15): 9682. DOI: 10.3390/ijerph19159682. PMC9367750 35955032

[24] Malterud K . Systematic text condensation: a strategy for qualitative analysis. Scand J Publ Health 2012; 40(8): 795–805. DOI: 10.1177/1403494812465030. 23221918

[25] Whitehead L . Enhancing the quality of hermeneutic research: decision trail. J Adv Nurs 2004; 45: 512–518. 15009354 10.1046/j.1365-2648.2003.02934.x

[26] Crabtree B Miller W . Doing qualitative research. 2nd ed. London, UK: Sage, 1999.

[27] Rushton CH Caldwell M Kurtz MCE . CE: moral distress: a catalyst in building moral resilience. Am J Nurs 2016; 116(7): 40–49. DOI: 10.1097/01.NAJ.0000484933.40476.5b. 27294668

[28] Varkey B . Principles of clinical ethics and their application to practice. Med Princ Pract 2021; 30(1): 17–28. DOI: 10.1159/000509119. 32498071 PMC7923912

[29] Jacobson N . A taxonomy of dignity: a grounded theory study. BMC Int Health Hum Right 2009; 9: 3. 10.1186/1472-698X-9-3 PMC2656457 19239684

[30] Chochinov HM . Dignity and the essence of medicine: the A, B, C, and D of dignity conserving care. BMJ 2007; 335(7612): 184–187. 17656543 10.1136/bmj.39244.650926.47 PMC1934489

[31] De Luca E Sena B Cataldi S . Supporting ethical end-of-life care during pandemic: palliative care team perspectives. Nurs Ethics 2023: 30: 570–584. DOI: 10.1177/09697330231153684. 36730007 PMC9899681

[32] Garcia JLA . Virtues and principles in biomedical ethics. J Med Philos 2020; 45(4-5): 471–503. DOI: 10.1093/jmp/jhaa013. 32726807

[33] Beauchamp TL Childress JF . Principles of biomedical ethics. 5th ed. Oxford, UK: Oxford University Press, 2001.

[34] Palmer JA Mccullough M Wormwood J , et al. Addressing clinician moral distress: implications from a mixed methods evaluation during Covid-19. PLoS One 2023; 18(9): e0291542. DOI: 10.1371/journal.pone.0291542. 37713379 PMC10503769

[35] Nilsen S Malterud K . What happens when the doctor denies a patient’s request? A qualitative interview study among general practitioners in Norway. Scand J Prim Health Care 2017; 35(2): 201–207. DOI: 10.1080/02813432.2017.1333309. 28581878 PMC5499321

[36] Nilsen S Malterud K Werner EL , et al. GPs’ negotiation strategies regarding sick leave for subjective health complaints. Scand J Prim Health Care 2015; 33(1): 40–46. DOI: 10.3109/02813432.2015.1001943. 25602364 PMC4377738

[37] Tong LK Zhu MX Wang SC , et al. The impact of caring for COVID-19 patients on nurse professional identity: a cross-sectional study using propensity score analysis. Front Public Health 2022; 10: 1066667. DOI: 10.3389/fpubh.2022.1066667. 36523574 PMC9745049

[38] Moula Z Horsburgh J Scott K , et al. The impact of Covid-19 on professional identity formation: an international qualitative study of medical students’ reflective entries in a Global Creative Competition. BMC Med Educ 2022; 22(1): 545. DOI: 10.1186/s12909-022-03595-1. 35836173 PMC9282904

[39] Karasek R Theorell J . Healthy work: stress, productivity and the reconstruction of working life. New York, NY: Basic Books, 1990.

[40] Wong CA Spence Laschinger HK . The influence of frontline manager job strain on burnout, commitment and turnover intention: a cross-sectional study. Int J Nurs Stud 2015; 52(12): 1824–1833. DOI: 10.1016/j.ijnurstu.2015.09.006. 26394531

[41] Han PKJ . Uncertainty in medicince. A framework for tolerance. Oxford, UK: Oxford University Press, 2021.

[42] Bringedal BH Rø KI Bååthe F , et al. Guidelines and clinical priority setting during the COVID-19 pandemic - Norwegian doctors' experiences. BMC Health Serv Res 2022; 22(1): 1192. DOI: 10.1186/s12913-022-08582-2. 36138400 PMC9503249

[43] Davidson PM Patch M . Time for a reset and recalibration: healthcare in the post COVID era. Int J Nurs Sci 2021; 8(2): 143–144. DOI: 10.1016/j.ijnss.2021.03.004. 33777478 PMC7979640

[44] Levoy K Foxwell A Rosa WE . Palliative care delivery changes during COVID-19 and enduring implications in oncology nursing: a rapid review. Curr Opin Support Palliat Care 2022; 16(3): 94–101. DOI: 10.1097/SPC.0000000000000603. 35929556 PMC9364779

[45] Muaygil R Aldekhyyel R AlWatban L , et al. Ethical uncertainty and COVID-19: exploring the lived experiences of senior physicians at a major medical centre. J Med Ethics 2023; 49(4): 275–282. DOI: 10.1136/jme-2022-108369. 36600609

[46] Ooi K . The pitfalls of overtreatment: why more care is not necessarily beneficial Asian Bioeth Rev 2020; 12(4): 399–417. DOI: 10.1007/s41649-020-00145-z. 33717342 PMC7747436

[47] Zimmerman JJ Harmon LA Smithburger PL , et al. Choosing wisely for critical care: the next five. Crit Care Med 2021; 49(3): 472–481. DOI: 10.1097/CCM.0000000000004876. 33555779

[48] Paladino J Mitchell S Mohta N , et al. Communication tools to support advance care planning and hospital care during the COVID-19 pandemic: a design process. Joint Comm J Qual Patient Saf 2021; 47(2): 127–136. DOI: 10.1016/j.jcjq.2020.10.005. PMC7584878 33191165

[49] Orstad S Fløtten Ø Madebo T , et al. The challenge is the complexity - a qualitative study about decision-making in advanced lung cancer treatment. Lung Cancer 2023; 183: 107312. DOI: 10.1016/j.lungcan.2023.107312. 37481888

[50] Zhang B Wright AA Huskamp HA , et al. Health care costs in the last week of life: associations with end-of-life conversations. Arch Intern Med 2009; 169(5): 480–488. DOI: 10.1001/archinternmed.2008.587. 19273778 PMC2862687

[51] Svantesson M de Snoo-Trimp JC Ursin G , et al. Important outcomes of moral case deliberation: a Euro-MCD field survey of healthcare professionals' priorities. J Med Ethics 2019; 45(9): 608–616. DOI: 10.1136/medethics-2018-104745. 31320403 PMC6817990

[52] Silverman HJ Kheirbek RE Moscou-Jackson G , et al. Moral distress in nurses caring for patients with Covid-19. Nurs Ethics 2021; 28(7-8): 1137–1164. DOI: 10.1177/09697330211003217. 33910406

[53] British Medical Association . Moral distress and moral injury: recognising and tackling it for UK doctors. London, England: British Medical Association, 2021, https://www.bma.org.uk/media/4209/bma-moral-distress-injury-survey-report-june-2021.pdf# (accessed 23 06 23).

[54] Jia Y Chen O Xiao Z , et al. Nurses’ ethical challenges caring for people with COVID-19: a qualitative study. Nurs Ethics 2021; 28(1): 33–45. DOI: 10.1177/0969733020944453. 32856534 PMC7653013

[55] Chang BKW Matthews P . How is COVID-19 changing the ways doctors make end-of-life decisions? J Med Ethics. 2022; 48: 941–947. DOI: 10.1136/medethics-2022-108268. 35879103

[56] Crico C Sanchini V Casali P , et al. Evaluating the effectiveness of clinical ethics committees: a systematic review. Med Health Care Philos 2021; 24(1): 135–151. DOI: 10.1007/s11019-020-09986-9. 33219898 PMC7910230

[57] Cunningham M Wells M . Qualitative analysis of 6961 free-text comments from the first national cancer patient experience survey in scotland. BMJ Open 2017; 7(6): e015726, DOI: 10.1136/bmjopen-2016-015726. PMC5734250 28619780

[58] Hansen MIT Haugen DF Sigurdardottir KR , et al., Factors affecting quality of end-of-life hospital care - a qualitative analysis of free text comments from the i-CODE survey in Norway. BMC Palliat Care 2020; 19(1): 98. DOI: 10.1186/s12904-020-00609-x. 32635903 PMC7341649

